# Apple Pomace as a Potential Source of Oxidative Stress-Protecting Dihydrochalcones

**DOI:** 10.3390/antiox13101159

**Published:** 2024-09-25

**Authors:** Ibrahim Rabeeah, Viktoria Gruber-Schmidt, Helen Murray, Negin Afsharzadeh, Renate Paltram, Silvija Marinovic, Hassan Zia, Olly Sanny Hutabarat, Mikko Hofsommer, Ana Slatnar, Christopher Schlosser, Karl Stich, Heidi Halbwirth, Manfred Gössinger, Christian Haselmair-Gosch

**Affiliations:** 1 Institute of Chemical, Environmental and Bioscience Engineering, Technische Universität Wien, Getreidemarkt 9, 1060 Vienna, Austriaheidrun.halbwirth@tuwien.ac.at (H.H.); 2 Department of Fruit Processing, Federal College and Institute for Viticulture and Pomology, Wiener Straße 74, 3400 Klosterneuburg, Austria; 3 Department of Horticultural Science and Landscape, University of Tehran, Karaj 31587-77871, Iran; 4 GfL—Gesellschaft für Lebensmittel-Forschung mbH, Landgrafenstraße 16, D-10787 Berlin, Germany; 5 Department of Agricultural Technology, Hassanudin University, Makassar 90245, Indonesia; 6 Department of Agronomy, Biotechnical Faculty, Chair for Fruit, Viticulture and Vegetable Growing, University of Ljubljana, Jamnikarjeva 101, SI-1000 Ljubljana, Slovenia

**Keywords:** apple, *Malus* × *domestica*, pomace, juice processing, phloridzin, dihydrochalcones, polyphenols, antioxidant activity, Ferric Reducing Antioxidant Power (FRAP)

## Abstract

Among fruits, the apple is unique for producing large amounts of the dihydrochalcone phloridzin, which, together with phloretin, its aglycone, is valuable to the pharmaceutical and food industries for its antidiabetic, antioxidant, and anticarcinogenic properties, as well as its use as a sweetener. We analysed the phloridzin concentration, total phenolic content, and antioxidant activity in the peel, flesh, seeds, juice, and pomace of 13 international and local apple varieties. In the unprocessed fruit, the seeds had the highest phloridzin content, while the highest total phenolic contents were mostly found in the peel. In processed samples, phloridzin and the total phenolic compounds especially were higher mostly in juice than in pomace. Moreover, the total phenolic content was much higher than the phloridzin content. Juice showed the highest antioxidant activity, followed by the peel and flesh. Across all samples, antioxidant activity did not directly correlate with phloridzin concentrations, suggesting that the antioxidant activity ascribed to phloridzin may need re-evaluation. In the Ferric Reducing Antioxidant Power (FRAP) assay, phloridzin only showed antioxidant activity at high concentrations when compared to its aglycone, phloretin. Considering the large amounts of apple juice produced by the juice industry, residual pomace is a promising source of phloridzin. For technical use, processing this phloridzin to phloretin would be advantageous.

## 1. Introduction

**Figure 1:**
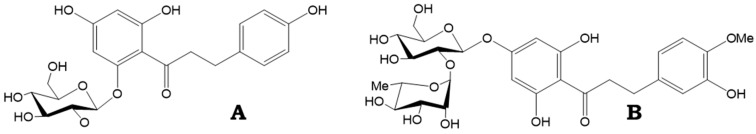
Chemical structure of phloridzin (**A**) and neohesperidin (**B**).

Dihydrochalcones are secondary metabolites in plants and are biochemically related to flavonoids. Their chemical structure is characterized by two aromatic rings linked by a saturated three-carbon bridge. They are notably abundant in the apple (*Malus × domestica*), but have also been identified in 28 other plant families [1]. In apples, specifically, phloridzin (phloretin 2′-*O*-glucoside) and its aglycone, phloretin (Figure 1A) are formed. Dihydrochalcones have garnered significant attention in research due to their beneficial effects on human health, including antioxidant, anti-inflammatory, anticancer, cholesterol-lowering, and antidiabetic effects [2,3,4].

Dihydrochalcones also hold high industrial value as artificial sweeteners (neohesperidin), usually derived from citrus (Figure 1B), which are commonly used in food and beverages, and provide a higher level of sweetness compared to sucrose and saccharin [5]. Phloretin is a flavouring substance granted GRAS status (generally recognized as safe) and is commercially available at a purity exceeding 90%. Despite lacking the sweetness of other dihydrochalcones, like neohesperidin, phloretin has the ability to increase existing sweetness, and also shows a strong masking effect against compounds with an unpleasant taste, such as caffeine, salicin, and quinine. It is also marketed for its potent antioxidant properties, making it of interest not only to the food industry but to the cosmetics industry [6].

Phloridzin was first described by Charles De Koninck in 1835 and, until the 1990s, it was assumed to be an apple-specific compound. In food analysis, it is therefore used as a lead substance for detecting adulteration of fruit juices with apple juice. With improved analytical instruments and increasingly lower detection limits, phloridzin was also described in other plant species, although in different concentrations [2,7]. Therefore, the apple must still be considered to be unique in terms of its high phloridzin formation. The vast amounts of phloridzin and phloretin in apples make it interesting as a unique source for extraction of these dihydrochalcones. Despite the comparatively large amounts of phloridzin accumulated in apples, its physiological role is largely unknown. It has been suggested that phloridzin is part of the apple’s pathogen defence mechanism, although it is present in fairly large quantities in all varieties, regardless of their susceptibility to disease, which does not support this hypothesis. However, recent findings suggest that degradation products of phloridzin are important, and that variation in the available enzyme spectrum and the velocity of the production of these products may be decisive in the disease tolerance of apple cultivars [8].

Phloridzin and phloretin are also important for humans for several reasons. Particularly interesting is their potential in treating diabetes [9], obesity [10], and stress hyperglycaemia [11]. Acting as an antidiabetic agent, phloridzin reduces the intestinal absorption of sugar by inhibiting the sodium-linked glucose transporters SGLT1 and SGLT2, leading to a decrease in blood glucose levels, which is particularly beneficial for patients with type 2 diabetes mellitus [9]. Furthermore, phloridzin serves as a natural antioxidant, effectively limiting the oxidation of proteins and lipids, and scavenging free radicals, thus playing a vital role in maintaining health [12]. Anticancer properties have also been demonstrated through the induction of cancer cell death via various pathways in different human cancer cell lines [13].

Apple fruit contains a wide range of bioactive compounds, including carbohydrates, fibres, polyphenols, organic acids, etc. All of these bioproducts have a significant impact as healthy dietary options. The phenolic compounds found in apples include flavanols and their polymeric forms, procyanidins and proanthocyanidins, water-soluble flavonoids (anthocyanins), and dihydrochalcones, such as phloridzin and its aglycon, phloretin. For dihydrochalcones, apples are a unique source in the human diet. It is one of the most commonly consumed fruits, with a worldwide harvest amount of around 95 million tonnes, with 18 million tonnes in Europe in the year 2022 [14]. Up to 75% of apples are consumed fresh. The remaining 25% are processed into a wide variety of products, including juices, ciders, and vinegar, as well as apple jam, frozen and dried apples, etc. During processing, 25–30% of the apple remains as byproduct pomace [15]. Since dihydrochalcones are mainly extracted from plant tissue [2]—whereas chemical or biotechnological synthesis are of little relevance [3]—this makes the pomace byproduct an important candidate source for extracting dihydrochalcones [16]. Dihydrochalcone composition is known to vary among different apple species/cultivars and plant parts [7,17] and is affected by processing techniques [18,19]. We therefore analysed the distribution of phloridzin in different fruit tissues, and along the processing line to apple juice. The samples included peels, flesh, seeds, pomace, fresh juice, and clarified juice; these were analysed for their physical and chemical characteristics, as well as for their antioxidant activity, phloridzin, and total phenolic content. To evaluate the impact of the cultivar further, 13 apple cultivars of international, as well as local, importance were analysed to depict a wider range of some less common genetic resources.

## 2. Materials and Methods

### 2.1. Plant Material

Samples from 13 apple *(Malus × domestica*) cultivars were used in this study. They included the following: cv. Elstar, cv. Roter Boskoop, cv. Kronprinz Rudolf, cv. Rubinette, cv. Roter Berlepsch, cv. Winterbanane, cv. Braeburn, cv. Goldparmäne, cv. Cox Orange, cv. Ilzer Rosenapfel, cv. Steirischer Maschanzker, cv. Gravensteiner, and cv. Topaz (Supplementary Table S1). Apples were harvested in the season of 2022 at the experimental orchard “Haschhof” of the Federal College and Institute for Viticulture and Pomology in Klosterneuburg, Lower Austria. Fruit and juice samples collected from each of these distinct apple cultivars were utilized for a comprehensive analysis of their physical and chemical characteristics to ensure the comparable maturity between samples (Supplementary Table S2).

Approximately 75 kg of each apple cultivar underwent a two-step juicing process. The fruits were washed, crushed with a grinding disc mill (Kreuzmayr, Wallern, Austria), and pressed with a Stossier belt press (Milteco GmbH, Anger, Austria). The resulting juice underwent enzymatic treatment with Fructozym P (2 mL/L, Fa. Erbslöh, Geisenheim, Germany), and clearing with bentonite (2 g/L NaCalit, Fa. Erbslöh, Geisenheim, Germany). Samples were collected directly after pressing (untreated juice) and after treatment (treated juice).

Thirty apple fruits from each cultivar were used for further testing. Weight measurements were conducted by weighing 10 apples per variant on a balance (EW 4200-2NM, Kern & Sohn GmbH, Balingen, Germany). Size measurements were taken for 10 apples per variant by measuring their widest point using standard digital callipers. Apples from each cultivar were divided into three parts: seeds, peel, and flesh. These parts were then homogenized and freeze-dried using a vacuum freeze-drying apparatus (Flexi-Dry™ MP, FTS SYSTEM, Stone Ridge, NY, USA) at −60 °C for 48 h. The freeze-dried samples were sealed and stored until further analysis.

### 2.2. Extraction of Phenols

The extraction process involved utilizing 80% methanol in a ratio of 1:10 (*w*/*v*). Extraction was facilitated through 1 h of sonication, followed by centrifugation at 1400 *× g* for 10 min at 4 °C. The supernatant was transferred to fresh reaction tubes and subsequently stored at −20 °C, preserving the samples for further analysis.

### 2.3. Analysis of Phloridzin Content

The phloridzin content was determined using a UHPLC system (Agilent 1290 Infinity II), consisting of 1290 Flexible Pump (G7104A), 1290 Multisampler (G7167B), 1290 Multicolumn Thermostat (G7116B), and 1290 DAD FS (G7117A). A ZORBAX^®^ Eclipse Plus C18, Rapid Resolution HD, 2.1 × 100 mm, 1.8 Micron (Agilent, Santa Clara, CA, USA) protected by the guard column UHPLC Guard ZORBAX^®^ Eclipse Plus C18, 2.1 × 5 mm, 1.8 Micron (Agilent, Santa Clara, CA, USA) was used for separation. The mobile phase consisted of ultrapure water with 0.1% formic acid (eluent A) and acetonitrile with 0.1% formic acid (eluent B). The gradient program was set as follows: 7.5% eluent B to 53% eluent B in 5.87 min, followed by 53% Eluent B to 95% eluent B in 3.75 min. A 5 min post-run was implemented to return to the initial conditions. The injection volume for all samples was 2 µL, with a flow rate of 0.5 mL/min, resulting in a total runtime of 15 min. Detection was performed at 290 nm, and spectra were acquired in the range of 190 nm to 640 nm.

### 2.4. Analysis of Total Phenolic Content (TPC)

The Folin–Ciocalteu assay described by Singleton and Rossi [20], and adapted by Juan and Jorge [21] for a 96-well microplate, was modified slightly further and used to determine the phenolic content of all parts of the 13 apple cultivars, with gallic acid as standard and water as blank. In brief, in a reaction tube, 600 µL of water was mixed with 10 µL of sample (apple parts and pomace extract, untreated and treated juice) and 50 µL of Folin–Ciocalteu reagent (diluted 1:1), and then incubated at room temperature for 8 min. Subsequently, 150 µL of 20% Na_2_CO_3_ and 190 µL of water were added to the mixture, followed by 30 min of incubation at 40 °C. The absorbance was then measured at 765 nm at room temperature. Results are presented as g gallic acid equivalents per kg of dry weight (DW).

### 2.5. Ferric-Reducing Antioxidant Power (FRAP) Assay

The FRAP assay, adapted from Benzie and Devaki [22], was conducted with a slight modification: Fresh FRAP reagent was prepared beforehand. In each well of a 96-well microplate, 10 µL of the sample (apple parts and pomace extract, untreated and treated juice) or standard solution phloridzin or phloretin (diluted in 95% EtOH) were added. Water was used as a blank. If necessary, the sample extracts were diluted with water for accurate readings. Then, 240 µL of FRAP reagent was added and the absorbance was measured at 593 nm at room temperature. Results are presented as g ascorbic acid equivalents per kg DW.

### 2.6. Statistical Analysis

A one-way analysis of variance (ANOVA) was performed using OriginPro software (Version 2023, OriginLab Corporation, Northampton, MA, USA). Statistical significance was set at the 0.05 level. Post hoc comparisons were conducted using Tukey’s HSD (honestly significant difference) test to evaluate differences between groups in all statistical analyses.

## 3. Results and Discussions

We first analysed apples as the raw material for apple juice. A thorough physical and chemical characterisation (Supplementary Table S2) confirmed the comparability of cultivars regarding ripeness. The concentration of phloridzin, as the main target compound, was analysed in the 13 cultivars by HPLC. Additionally, the total phenolic content was estimated by a spectrometer using the Folin–Ciocalteu method. Subsequently, we analysed the phloridzin and total phenolic content in the juice, as well as the byproduct pomace. This investigation led to interesting insights surrounding the antioxidant capacity of phloridzin.

### 3.1. Phloridzin and Total Phenolic Content in Different Apple Fruit Parts

**Figure 2:**
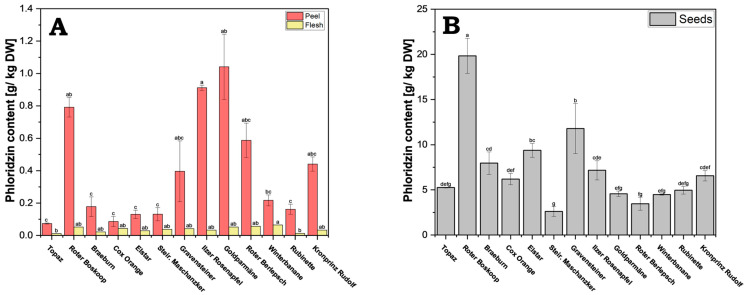
Phloridzin content of apple. Because of the large concentration differences, results are presented separately for peel and flesh (**A**), and for seeds (**B**). Results are shown as g phloridzin per kg dry weight (DW). The HPLC measurements were performed in triplicates. Different letters (a, b, c, etc.) between cultivars indicate statistically significant differences (*p* < 0.05).

Apples from each cultivar were separated into their respective components: seeds, flesh, and peel. These components were homogenized and freeze-dried. Subsequently, the phenolic compounds were isolated using sonication-assisted methanol extraction. The phloridzin content showed a substantial variation among the different cultivars and different fruit parts (Figure 2A,B and Supplementary Table S3).

Within the individual cultivars, the seeds contained significantly more phloridzin compared to flesh (roughly 60–400-fold) and peel (5–70-fold). There was also a very large variation in concentrations in peels from the different cultivars, with a significant but lesser variation seen in seeds across the cultivars. The flesh, which is the largest share of the fruit, generally showed lower concentrations and not much variation between the cultivars.

From all cultivars tested, cv. Roter Boskoop showed the highest phloridzin concentration in the seeds (19.84 g/kg DW); it also showed very high values in the peel. While the concentration in seeds was higher than that of peels in all cultivars, the ratio of seed to peel concentrations varied considerably, with cv. Elstar showing a ratio of roughly 72:1, and that of cv. Goldparmäne being only 4:1. Cv. Steirischer Maschanzker showed the lowest concentration in seeds (2.62 g/kg DW), and cv. Topaz had overall very low concentration, with its concentration in the peel being the lowest of all the cultivars (0.07 g/kg DW).

Interestingly, although cv. Goldparmäne had the highest concentration in the peel (1.04 g/kg DW), it had only an average concentration in the seeds. In addition to cv. Goldparmäne, cv. Gravensteiner, cv. Kronprinz Rudolf, cv. Roter Berlepsch and cv. Ilzer Rosenapfel all had relatively high phloridzin concentrations in the peel (0.40–0.91 g/kg DW). In the flesh, cv. Winterbanane (0.064 g/kg DW) exhibits the highest value in comparison to cv. Rubinette (0.013 g/kg DW) and cv. Topaz (0.012 g/kg DW), which hold the lowest values.

Total phenolic content (TPC) was estimated using the Folin–Ciocalteu colorimetric method. This gave us some insight into the approximate ratios of phloridzin to TPC, which are very low in the peel and flesh parts of the apple.

**Figure 3:**
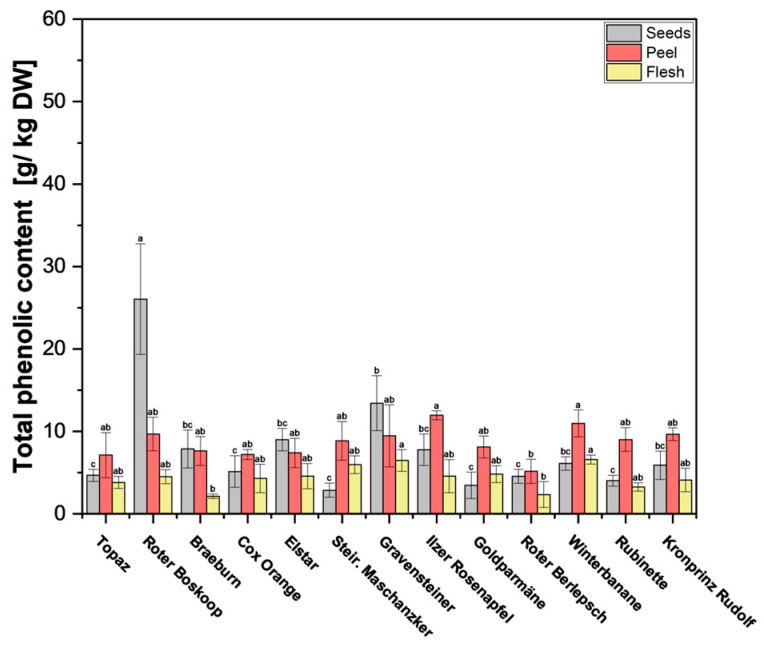
Total phenolic content (TPC) of apple fruit parts peel, flesh, and seeds. The measurements were performed in triplicates. Results are shown as g gallic acid equivalent per kg of dry weight (DW). Different letters (a, b, c) between cultivars indicate statistically significant differences (*p* < 0.05).

In previous studies [23,24], it was found that the polyphenol content in apple peels surpasses that found in the flesh, which is supported by our findings (Figure 3 and Supplementary Table S4). Although the flesh showed significantly lower TPC across all cultivars, the difference here was smaller than with phloridzin, with TPC in the flesh fairly consistent across all cultivars. Of particular interest are the seeds, some of which have notably high values. Of the 13 cultivars tested, cv. Roter Boskoop seeds showed significantly higher content (28.8 g/kg DW), with the rest of the seeds of the remaining cultivars ranging between 2.9 and 13.4 g/kg DW. In most cultivars, TPC was quite similar in the peel when compared with the seeds, which stands in contrast to the phloridzin content, which was significantly higher in seeds across all cultivars. Only cv. Roter Boskoop, cv. Braeburn, cv. Elstar, and cv. Gravensteiner showed significantly lower TPC in the peel compared with the seeds (Supplementary Table S4) In the cultivars tested here, phloridzin accounted for, on average, 60% of TPC in seeds, but only around 5% in the peel, and 1% of TPC in the flesh. The values found in the seeds are a bit lower than in previous studies, such as Awad et al. [23], where phloridzin in the seeds contributed to around 98% of the total flavonoids, and Lu et al. [25], who report that phloridzin constituted around 75% of the total polyphenols.

**Figure 4:**
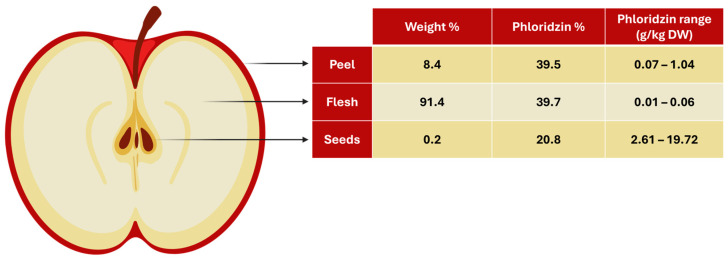
Illustration showing the percentage of weight and phloridzin for each part of a dried apple (seeds, flesh, peel), as well as the phloridzin range for each. (Illustration created with BioRender.com).

When examining the contribution of each part to the total dried apple weight in terms of percentage, the breakdown is as follows: flesh 91.4%, peel 8.4%, and seeds 0.2%. (Figure 4). As a result, although the phloridzin concentrations in the different parts vary greatly, the distribution of phloridzin in total amounts is fairly even, with the flesh contributing roughly 40%, the peel 40%, and the seeds 20%. This suggests that the entire apple can serve as a valuable source of phloridzin.

### 3.2. Phloridzin and Total Phenolic Content from Apple Juice

The apple juice samples were categorized into three distinct samples: untreated juice, treated juice, and the byproduct pomace. The untreated juice was collected immediately after pressing, while the treated juice was collected after clearing with bentonite and pectinase. The pomace was homogenized and freeze-dried, followed by subsequent extraction of polyphenols using the same method as described for the apple parts. Both the pomace extracts and juice were directly utilized for HPLC quantification.

**Figure 5:**
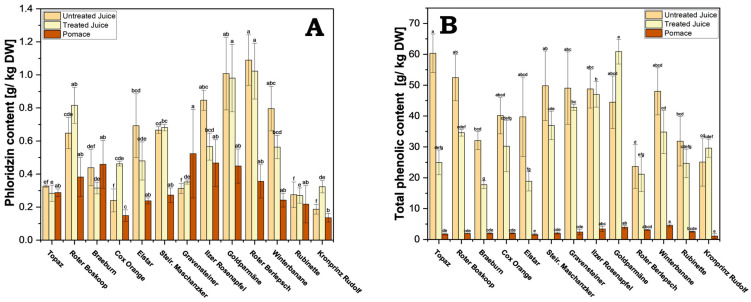
(**A**) Phloridzin content of the processed juice samples (treated and untreated) and pomace. Results are shown as g phloridzin per kg dry weight (DW); (**B**) total phenolic content for processed juice (treated and untreated) and pomace. Results are shown as g gallic acid equivalents per kg dry weight (DW). The HPLC measurements were performed in triplicates. Different letters (a, b, c, etc.) between cultivars indicate statistically significant differences (*p* < 0.05).

Phloridzin distribution in the juices and pomace varied greatly between cultivars. Phloridzin was detected across all components of the processed apple juice, with higher content predominantly observed in the juices across various cultivars (Figure 5A and Supplementary Table S3). Interestingly, phloridzin content did not vary significantly between treated and untreated juices. In the case of untreated juice, cv. Roter Berlepsch exhibited the highest content (1.09 g/kg), followed by cv. Goldparmäne (1.01 g/kg), while the lowest values were recorded for cv. Kronprinz Rudolf (0.19 g/kg).

Interestingly, although phloridzin is water soluble, significant amounts were found in the pomace, with a per cultivar comparison revealing an average of 67% as much phloridzin in pomace when compared with the untreated juice of the same cultivar. This means a considerable amount of the total phloridzin is left behind in the pomace during processing. Roughly a 0.3-fold (cv. Winterbanane) to 1.6-fold (cv. Gravensteiner) amount of phloridzin can be found in pomace compared with juice in general. This makes pomace a viable source of significant amounts of phloridzin. On average, across all cultivars, a yield of 0.32 g of phloridzin per kg DW of pomace can be expected.

In contrast to phloridzin, the difference in TPC between juice and pomace was much greater (Figure 5B and Supplementary Table S4). This may be due to a vastly greater solubility of many polyphenols when compared with that of phloridzin, and thus would be displaced into the juice during pressing. As observed with phloridzin, no significant differences in TPC between treated and untreated juice were found, except with cv. Topaz, cv. Roter Boskoop, cv. Braeburn, and cv. Elstar (Supplementary Table S4). The highest amounts in untreated juice were found in cv. Topaz (60.4 g/kg DW), followed by cv. Roter Boskoop (52.4 g/kg DW), and cv. Steirischer Maschanzker (49.8 g/kg DW). The lowest amounts belong to cv. Roter Berlepsch (23.7 g/kg DW) and cv. Kronprinz Rudolf (25.1 g/kg DW).

### 3.3. Ferric Reducing Antioxidant Power (FRAP) Assay

Given the much-touted antioxidant value of phloridzin and phloretin, we were also interested in assessing antioxidant activity. We used the semi-automated Ferric Reducing Antioxidant Power (FRAP) method, described by Benzie and Devaki [22]. The results of the assay are expressed as g of ascorbic acid equivalents per kg dry weight. In contrast to previous studies, such as Ozyilmaz and Gunay [26], significant increases in antioxidant power after treatment with bentonite and pectinase was found only in a few cases.

**Figure 6:**
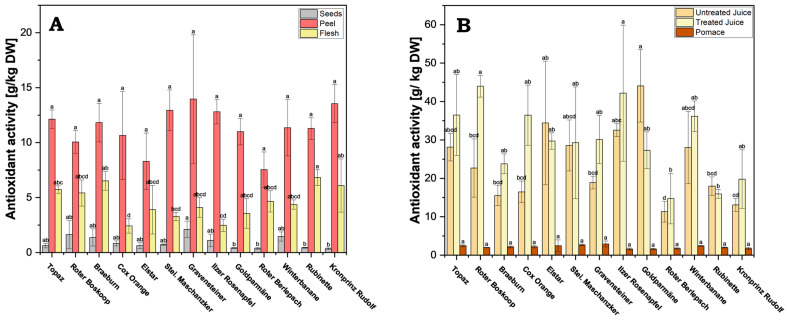
Ferric Reducing Antioxidant Power (FRAP): (**A**) processed juice samples (treated and untreated) and pomace; (**B**) apple fruit parts peel, flesh and seeds. Results are shown as g ascorbic acid equivalents per kg dry weight (DW) of extract or juice. FRAP measurements were performed in triplicates. Different letters (a, b, c, etc.) between cultivars indicate statistically significant differences (*p* < 0.05).

Across all cultivars, antioxidant activity was significantly higher in the peel, ranging between 7.55 g/kg DW (cv. Roter Berlepsch) and 13.97 g/kg DW (cv. Gravensteiner) (Figure 6A and Supplementary Table S5). For example, the antioxidant activity in the flesh ranged from 2.42 g/kg DW (cv. Cox Orange) to 6.82 g/kg DW (cv. Rubinette), and in the seeds only from 0.36 g/kg DW (cv. Kronprinz Rudolf) to 2.11 g/kg DW (cv. Gravensteiner). The apple peel functions as a protective layer of the fruit, and naturally it will contain active compounds to combat diseases and oxidative stress, which explains the high FRAP value compared to that of the flesh or seeds [27].

The analysis of the data reveals vastly lower antioxidant activity in pomace across all cultivars, when compared to both treated and untreated juice, ranging between 1.59 g/kg DW (cv. Goldparmäne) and 2.93 g/kg DW (cv. Gravensteiner) (Figure 6B and Supplementary Table S5). By comparison, the activity in untreated juice ranged from 11.34 g/kg DW (cv. Roter Berlepsch) to 44.07 g/kg DW (cv. Goldparmäne), and in treated juice from 14.74 g/kg DW (cv. Roter Berlepsch) to 43.99 g/kg DW (cv. Roter Boskoop). This indicates that most of the antioxidant active compounds are transferred in the juice fraction during processing.

**Figure 7:**
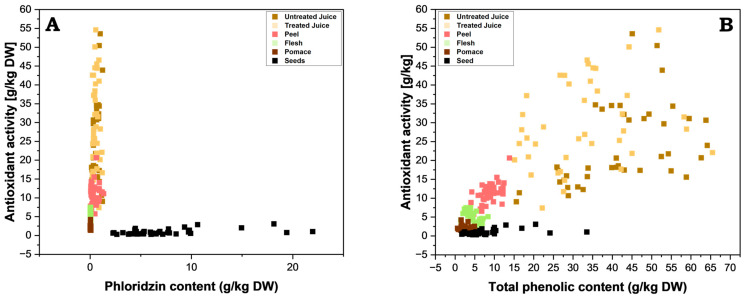
Juxtaposition of antioxidant activity (FRAP) and the content of (**A**) phloridzin and (**B**) total phenolic compounds. Individual graphs for seeds, peels, flesh, pomace, and juices are available in the Supplementary Figures S1 and S2.

Also noteworthy is that the very high phloridzin content observed in apple seeds did not correlate to high antioxidant activities. This combination of high phloridzin content and low antioxidant activity contradicts the consensus amongst studies, where phloridzin is often cited as a strong antioxidant [28,29,30,31], even when considering that different test systems (e.g., FRAP vs. others) may produce slightly different results [28,32,33]. To investigate this further, the FRAP values were plotted against the phloridzin content and the TPC values for each part of the apple, the juices, and pomace (Figure 7; separate plots can be found in the Supplementary Figures S1 and S2). No clear correlation between the antioxidant activity and the TPC or phloridzin content was found throughout all apple parts. However, a tendential correlation was visible and could form the basis for further investigation. For phloridzin, this can be explained by the lack of a double bond between C2 and C3 and vicinal hydroxy groups in the B-ring, all of which are prominent structural elements relevant for antioxidant activity in flavonoids [34]. A further important factor is the meta dihydroxy moiety in the A-ring in combination with the 4-oxo group, whereas in phloridzin the vicinal hydroxy group is glycosylated (position 2′), and is therefore protected against an oxidative attack [34].

**Figure 8:**
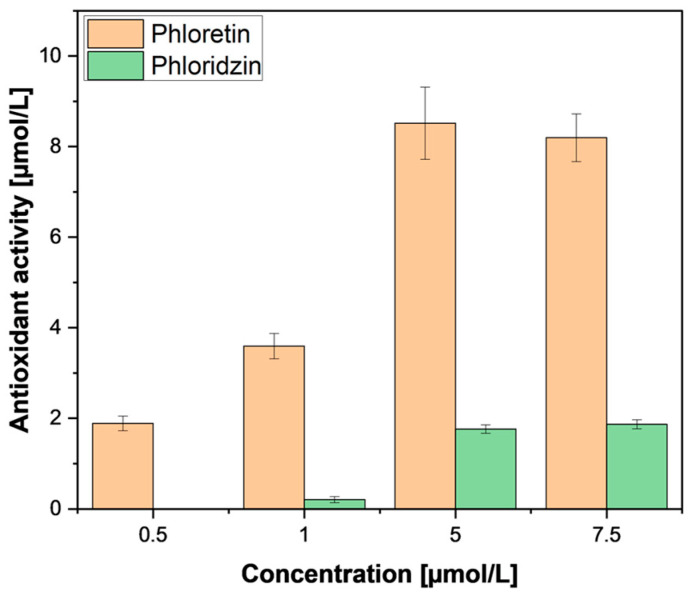
FRAP assay of phloridzin and phloretin standard compounds. FRAP measurements were performed in triplicates. Results are shown as µmol ascorbic acid equivalents per litre.

Therefore, further investigation was performed using pure phloretin and phloridzin standards from a commercial supplier (Merck, St. Louis, MO, USA). In accordance with our samples, purified phloridzin also did not exhibit any pronounced antioxidant activity (Figure 8) compared to phloretin; this was also found by Zielinska et al. [29]. This low activity in the relatively high presence of phloridzin leads us to conclude that the antioxidant activity ascribed to phloridzin may need re-evaluation. The observed antioxidant activity could be attributed to the other phenolic compounds extracted. Meanwhile, the weak activity of phloretin could be explained by the removal of the sugar moiety, freeing a hydroxyl group which is important for antioxidant activity [35].

Vitamin C was considered as another possible source of antioxidant capacity, as it is known to afford a high level of protection from oxidation. However, apple juice has relatively low vitamin C levels [36]. The juice samples were nonetheless tested for their vitamin C content using a novel HILIC-MS/MS method [37]. The results of the tests confirmed a low vitamin C content, with only the oxidized form, dehydroascorbic acid, found in almost all cultivars, the exception being cv. Rubinette and cv. Gravensteiner, which contained 211.5 and 11.3 mg/L of ascorbic acid per L of juice, respectively (Supplementary Table S6). This leads us to the conclusion that other factors may be at play.

## 4. Conclusions

Dihydrochalcones, such as phloridzin and phloretin, which are known for their beneficial health effects, are present in vast amounts in apples. Phloridzin content in the apple was found to be higher in the seeds and peel than in the flesh; whereas during the juicing process, a lot of the total phloridzin content remains in the pomace. TPC content did not vary that much among different apple tissues, as was found for phloridzin, but most of the TPC is present in the juice and not in the pomace. The antioxidant activity is mainly present in the peel and comparably low in the seeds. During the juicing process, antioxidant components can mainly be found in the juice and not in the pomace. In general, phloridzin content, TPC and antioxidant activity can also be affected greatly by the cultivar. Taking into account the high level of remaining phloridzin in the pomace, together with the use of environmentally friendly extraction methods, this could make processing byproducts an interesting candidate source for the future extraction of dihydrochalcones.

The wealth of data presented here provide an excellent opportunity to re-evaluate the purported antioxidant activity of phloridzin. However, these data suggest that this antioxidant activity is overstated. Having said that, these high concentrations of phloridzin could serve as a valuable source for phloretin production [38], which would lead to a marked increase in antioxidative capacity.

## Data Availability

The original data presented in the study are openly available in the research data repository “TU Wien Research Data” under the following https://doi.org/10.48436/6r0qw-s1y20 (accessed on 27 August 2024).

## Supplementary Materials

The following supporting information can be downloaded at: https://www.mdpi.com/article/10.3390/antiox13101159/s1, Table S1: Origin and characteristics of the apple cultivars. Table S2: Physical and chemical characteristics of the apple cultivars. Table S3: Phloridzin content of processed juice and solid apple parts. Table S4: Total phenolic content of the processed juice and solid apple parts. Table S5: Ferric Reducing Antioxidant Power (FRAP) result for the processed juice and solid apple parts. Table S6: Vitamin C and dehydroascorbic acid content for the processed apple juice. Figure S1: Juxtaposition of antioxidant activity (FRAP) and the content of phloridzin in all processed juice and solid apple parts sample. Figure S2: Juxtaposition of antioxidant activity (FRAP) and the total phenolic content in all processed juice and solid apple parts sample. References [39,40,41,42] are cited in the Supplementary Materials.
