# Supplementary material for: Apple Pomace as a Potential Source of Oxidative Stress-Protecting Dihydrochalcones
Source: Antioxidants (Basel). 2024 Sep 25;13(10):1159. doi: 10.3390/antiox13101159 (PMC11505204; doi:10.3390/antiox13101159)
Supplement: Supplementary file 1 [file antioxidants-13-01159-s001.zip › antioxidants-3204542-supplementary.pdf]

Supplementary Table S1. Origin and characteristics of the apple cultivars.

Cultivar	Origin	Characteristics	Reference
Braeburn	NZ	late ripening, medium to large size, yellowish flesh, firm, but juicy, balanced sour-sweet taste, yellow-green and half brown-red skin	[1]
Cox Orange	FR	midseason, medium to large size, yellowish flesh, medium firm and juicy, sweet taste with a slight acidity, yellowish, green to red skin	[1]
Elstar	NL	midseason, medium size, yellowish flesh, medium firm, sweet and sour taste in a pleasant ratio. yellow skin, partially red	[1]
Goldparmäne	FR	midseason, medium size, yellowish flesh, delicately sweet and sour taste, reddish skin with a yellow base color	[1]
Gravensteiner	IT (?)	origin not exactly defined, early ripening, medium size, yellowish flesh, intensive sweet-sour taste. flamed red skin with a yellow base color	[1]
Ilzer Rosenapfel	AT	midseason, small to medium size, white to greenish white, flesh, firm but juicy, mild sweet-sour taste, pink to dark red skin	[2]
Kronprinz Rudolf	AT	early ripening, small to medium size, white flesh, juicy, mildly sour, moderately sweet taste, greenish skin with intensive red parts	[3]
Roter Berlepsch	DE	midseason, medium size, yellowish flesh, firm and very juicy, striped or marbled yellow to reddish brown skin	[3]
Roter Boskoop	NL	midseason, big size, yellowish flesh, firm, pleasantly sour taste, yellow-green skin with brick red marbling	[1]
Rubinette	CH	late ripening, big size, yellowish flesh, medium firm, juicy, sweet aroma with noticeable acidity, reddish to yellow skin	[1]
Steirischer Maschanzker	AT	midseason, small to medium size, yellowish flesh, firm but juicy, sweet taste, green to yellow skin	[4]
Topaz	CZ	midseason, medium size, yellowish flesh, firm, juicy, sweet taste with remarkable acid, yellow skin with striped red parts	[1]
Winterbananenapfel	US	midseason, medium size, yellowish flesh, juicy, mildly sour-sweet, yellow-green skin	[3]

Supplementary Table S2. Physical and chemical characteristics of the apple cultivars.

Cultivar	pH	Titratable Acidity (Malic Acid Equivalents g.L^{-1})	Weights (g)	Size (mm)	Brix (°Bx)	Starch (Streif Index)	Firmness
Topaz	3.10	10.39	190.69	79.67	15.2	7.0	6.86
Roter Boskoop	2.75	13.13	261.84	85.47	17.4	6.6	7.23
Braeburn	3.33	7.57	160.39	75.85	13.4	6.8	8.86
Cox Orange	3.34	9.85	172.54	76.52	16.9	7.2	5.40
Elstar	3.26	7.84	126.41	68.15	15.0	8.2	5.23
Steir. Maschanzker	3.30	7.84	113.57	65.55	15.5	7.8	8.27
Gravensteiner	3.07	6.77	187.17	77.87	13.4	9.8	4.37
Ilzer Rosenapfel	3.26	8.71	98.08	62.60	15.4	7.4	8.56
Goldparmäne	3.26	9.31	139.23	69.88	17.1	9.2	6.59
Roter Berlepsch	3.13	10.05	120.48	75.80	14.8	7.0	6.92
Winterbanane	3.27	5.83	244.71	87.53	14.6	8.4	6.45
Rubinette	3.21	9.05	132.71	68.20	18.2	8.2	6.37
Kronprinz Rudolf	3.21	7.17	122.89	68.86	13.8	8.7	6.31

The pH-values were determined using a pH-meter (MultilineP4 with SenTix 41-3, WTW). Titratable acidity was determined by titration to an endpoint of pH-value 7.0 (0.1 N NaOH Titrisol, sodium hydroxide solution for 1000 mL, HC872021, Merck) and then multiplied by the acidity factor of malic acid (0.67) to express acidity in g/L malic acid equivalents). Soluble solids (sugar content, °Brix) were measured using a handheld refractometer (N-20, Brix 0~20%, ATAGO). The starch iodine test was employed to assess the apples' maturity. The Streif index was calculated as [Firmness/ (Brix° X Starch index)] (DeLong et al., DOI:10.21273/HORTSCI.34.7.1251). Firmness values were obtained using a penetrometer (Mecmesin, AFG 500N, stamp 11.55 mm) measuring 10 fresh apples on 2 sides (20 measurements total).

Supplementary Table S3. Phloridzin content of processed juice and solid apple parts. The HPLC measurements were performed in triplicates. Results are shown as g phloridzin per kg dry weight (DW). Different letters (a, b, c, etc.) indicate statistically significant differences ($p < 0.05$). **A:** statistical difference between cultivars in a column. **B**: statistical difference between processed juice sample for the same cultivar in a row. **C:** statistical difference between solid apple parts for the same cultivar in a row.

A

Cultivar	Untreated Juice	Treated Juice	Pomace	Peel	Flesh	Seeds
Topaz	0.327 ±0.007ef	0.284 ±0.050e	0.287 ±0.023ab	0.074 ±0.003f	0.012 ±0.003b	5.239 ±0.060defg
Roter Boskoop	0.648 ±0.094cde	0.816 ±0.109ab	0.382 ±0.118ab	0.792 ±0.060ab	0.052 ±0.020ab	19.837 ±1.945a
Braeburn	0.439 ±0.110def	0.316 ±0.036de	0.459 ±0.140ab	0.178 ±0.060ef	0.022 ±0.020ab	7.961 ±1.250cd
Cox Orange	0.240 ±0.069f	0.462 ±0.014cde	0.149 ±0.035c	0.086 ±0.030f	0.043 ±0.030ab	6.195 ±0.630def
Elstar	0.693 ±0.194bcd	0.479 ±0.115cde	0.238 ±0.020ab	0.130 ±0.030f	0.029 ±0.015ab	9.376 ±0.770bc
Steir. Maschanzker	0.666 ±0.020cd	0.682 ±0.020bc	0.272 ±0.040ab	0.131 ±0.040f	0.038 ±0.003ab	2.615 ±0.541g
Gravensteiner	0.313 ±0.031f	0.352 ±0.012de	0.523 ±0.270a	0.396 ±0.190cde	0.042 ±0.020ab	11.786 ±2.770b
Ilzer Rosenapfel	0.847 ±0.061abc	0.566 ±0.082bcd	0.467 ±0.140ab	0.912 ±0.020a	0.033 ±0.003ab	7.168 ±1.060cde
Goldparmäne	1.008 ±0.220ab	0.981 ±0.2004a	0.449 ±0.100ab	1.041 ±0.200a	0.052 ±0.030ab	4.569 ±0.290efg
Roter Berlepsch	1.090 ±0.153a	1.023 ±0.169a	0.356 ±0.100ab	0.588 ±0.100bc	0.057 ±0.020ab	3.467 ±0.722fg
Winterbanane	0.796 ±0.135abc	0.564 ±0.071bcd	0.242 ±0.040ab	0.217 ±0.034def	0.064 ±0.020a	4.481 ±0.070efg
Rubinette	0.275 ±0.078f	0.270 ±0.046e	0.218 ±0.110ab	0.162 ±0.031ef	0.013 ±0.001b	4.954 ±0.430defg
Kronprinz Rudolf	0.187 ±0.029f	0.323 ±0.038de	0.136 ±0.030b	0.441 ±0.044cd	0.033 ±0.020ab	6.565 ±0.570cdef

B

Cultivar	Untreated Juice	Treated Juice	Pomace
Topaz	0.327 ±0.010a	0.284 ±0.050a	0.287 ±0.023a
Roter Boskoop	0.648 ±0.090ab	0.816 ±0.109a	0.382 ±0.118b
Braeburn	0.439 ±0.110a	0.316 ±0.036a	0.459 ±0.140a
Cox Orange	0.240 ±0.040b	0.462 ±0.014b	0.149 ±0.035b
Elstar	0.693 ±0.190a	0.479 ±0.115ab	0.238 ±0.020b
Steir. Maschanzker	0.666 ±0.020a	0.682 ±0.020a	0.272 ±0.040b
Gravensteiner	0.313 ±0.030a	0.352 ±0.012a	0.523 ±0.270a
Ilzer Rosenapfel	0.847 ±0.061a	0.566 ±0.082b	0.467 ±0.140b
Goldparmäne	1.008 ±0.220a	0.981 ±0.200a	0.449 ±0.100b
Roter Berlepsch	1.090 ±0.150a	1.023 ±0.169a	0.356 ±0.100b
Winterbanane	0.796 ±0.135a	0.564 ±0.071b	0.242 ±0.040c
Rubinette	0.275 ±0.080a	0.270 ±0.046a	0.218 ±0.110a
Kronprinz Rudolf	0.187 ±0.030b	0.323 ±0.038a	0.136 ±0.030b

C

Cultivar	Peel	Flesh	Seeds
Topaz	0.074 ±0.003b	0.012 ±0.003b	5.239 ±0.060a
Roter Boskoop	0.792 ±0.060b	0.052 ±0.020b	19.837 ±1.945a
Braeburn	0.178 ±0.060a	0.022 ±0.020b	7.961 ±1.250b
Cox Orange	0.086 ±0.030b	0.043 ±0.030b	6.195 ±0.630a
Elstar	0.130 ±0.030b	0.029 ±0.015b	9.376 ±0.770a
Steir. Maschanzker	0.131 ±0.040b	0.038 ±0.003b	0.2615 ±0.541a
Gravensteiner	0.396 ±0.190b	0.042 ±0.020b	11.786 ±2.770a
Ilzer Rosenapfel	0.912 ±0.020b	0.033 ±0.003b	7.168 ±1.060a
Goldparmäne	1.041 ±0.200b	0.052 ±0.030c	4.569 ±0.290a
Roter Berlepsch	0.589 ±0.100b	0.057 ±0.020b	3.467 ±0.722a
Winterbanane	0.217 ±0.030c	0.064 ±0.020b	4.481 ±0.070a
Rubinette	0.162 ±0.030b	0.013 ±0.001b	4.954 ±0.430a
Kronprinz Rudolf	0.441 ±0.040b	0.033 ±0.020b	6.565 ±0.570a

Supplementary Table S4. Total phenolic content of the processed juice and solid apple parts. The measurements were performed in triplicates. Results are shown as g gallic acid equivalent per kg of dry weight (DW). Different letters (a, b, c, etc.) indicate statistically significant differences ($p < 0.05$). **A:** statistical difference between cultivars in a column. **B**: statistical difference between processed juice sample for the same cultivar in a row. **C:** statistical difference between solid apple parts for the same cultivar in a row.

A

Cultivar	Untreated Juice	Treated Juice	Pomace	Peel	Flesh	Seeds
Topaz	60.4 ±6.3a	25.0 ±4.0defg	1.8 ±0.3de	7.3 ±2.7ab	3.8 ±0.7ab	4.7 ±0.7c
Roter Boskoop	52.4 ±7.5ab	34.6 ±1.1cdef	2.0 ±0.1cde	9.7 ±2.1ab	4.5 ±0.9ab	28.8 ±6.6a
Braeburn	32.0 ±2.9bcd	17.8 ±1.3g	2.1 ±0.3cde	7.7 ±1.6ab	2.1 ±0.3b	7.9 ±2.3bc
Cox Orange	40.2 ±5.9abcd	30.2 ±8.3cdefg	2.1 ±0.2cde	7.3 ±0.ab	4.3 ±1.7ab	5.1 ±1.9c
Elstar	39.8 ±12.9abcd	18.9 ±3.1fg	1.7 ±0.4e	7.7 ±1.9ab	4.6 ±1.5ab	9.0 ±1.4bc
Steir. Maschanzker	49.8 ±11.3ab	37.0 ±4.6cde	2.1 ±0.4cde	9.0 ±2.2ab	6.0 ±1.1ab	2.9 ±0.9c
Gravensteiner	49.1 ±11.8abc	42.8 ±1.0bc	2.5 ±0.9cde	9.5 ±3.8ab	6.5 ±1.3a	13.4 ±3.4b
Ilzer Rosenapfel	48.8 ±6.2abc	47.0 ±4.1b	3.4 ±0.9ab	12.0 ±0.4a	4.6 ±2.0ab	7.8 ±1.9bc
Goldparmäne	44.5 ±8.5abcd	60.9 ±4.0a	4.1 ±0.5a	8.3 ±1.5ab	4.8 ±1.0ab	3.5 ±1.6c
Roter Berlepsch	23.7 ±7.1d	21.2 ±5.6efg	3.1 ±0.2abcd	5.2 ±1.4b	2.3 ±1.6b	4.5 ±0.9c
Winterbanane	48.0 ±7.7abcd	34.8 ±7.0cd	4.5 ±0.5a	11.1 ±1.6a	6.6 ±0.6a	6.1 ±0.8bc
Rubinette	31.8 ±8.0bcd	24.7 ±4.6cdefg	2.6 ±0.2bcde	9.2 ±1.6ab	3.3 ±0.5ab	4.1 ±0.7c
Kronprinz Rudolf	25.1 ±7.8cd	29.6 ±2.9cdef	1.1 ±0.8d	9.9 ±0.8ab	4.1 ±1.4ab	5.9 ±1.7bc

B

Cultivar	Untreated Juice	Treated Juice	Pomace
Topaz	60.4 ±6.3a	25.0 ±4.0b	1.8 ±0.3c
Roter Boskoop	52.4 ±7.5a	34.6 ±1.1b	2.0 ±0.1c
Braeburn	32.0±2.9a	17.8 ±1.3b	2.1 ±0.2c
Cox Orange	40.2 ±5.9a	30.2 ±8.3a	2.1 ±0.2b
Elstar	39.8 ±12.9a	18.9 ±3.1b	1.7 ±0.4b
Steir. Maschanzker	49.8 ±11.3a	37.0 ±4.6a	2.1 ±0.4b
Gravensteiner	49.1 ±11.8a	42.8 ±1.0a	2.5 ±0.9b
Ilzer Rosenapfel	48.8 ±6.2a	47.0 ±4.2a	3.5 ±0.9b
Goldparmäne	44.5 ±8.5b	60.9 ±4.0a	4.1 ±0.5c
Roter Berlepsch	23.7 ±7.1a	21.2 ±5.6a	3.1 ±0.2b
Winterbanane	48.0 ±7.7a	34.8 ±7.0a	4.5 ±0.5b
Rubinette	31.8 ±8.0a	24.7 ±4.6a	2.6 ±0.2b
Kronprinz Rudolf	25.1 ±7.8a	29.6 ±3.0a	1.1 ±0.8b

C

Cultivar	Peel	Flesh	Seeds
Topaz	7.3 ±2.8a	3.9 ±0.8a	4.7 ±0.7a
Roter Boskoop	9.7 ±2.1b	4.6 ±0.8b	28.8 ±6.6a
Braeburn	7.7 ±1.6a	2.2 ±0.3b	7.9 ±2.3a
Cox Orange	7.3 ±0.5a	4.4 ±1.8a	5.1 ±1.9a
Elstar	7.7 ±1.9ab	4.7 ±1.5b	9.1 ±1.4a
Steir. Maschanzker	9.0 ±2.2a	6.2 ±1.0ab	2.9 ±0.9b
Gravensteiner	9.5 ±3.8a	6.7 ±1.5a	13.4 ±3.4a
Ilzer Rosenapfel	12.0 ±0.4a	4.7 ±2.1b	7.8 ±1.9b
Goldparmäne	8.3 ±1.5a	5.0 ±1.1ab	3.5 ±1.6b
Roter Berlepsch	5.2 ±1.4a	2.4 ±1.6a	4.5 ±0.9a
Winterbanane	11.1 ±1.6a	6.7 ±0.4b	6.1 ±0.8b
Rubinette	9.2 ±1.6a	3.4 ±0.5b	4.1 ±0.7b
Kronprinz Rudolf	9.9 ±0.8a	4.2 ±1.5b	5.9 ±1.7b

Supplementary Table S5. Ferric Reducing Antioxidant Power (FRAP) result for the processed juice and solid apple parts. the measurements were performed in triplicates. Results are shown as g gallic acid equivalent per kg of dry weight (DW). Different letters (a. b. c. etc.) indicate statistically significant differences ($p < 0.05$). **A:** statistical difference between cultivars in a column. **B**: statistical difference between processed juice sample for the same cultivar in a row. **C:** statistical difference between solid apple parts for the same cultivar in a row.

A

Cultivar	Untreated Juice	Treated Juice	Pomace	Peel	Flesh	Seeds
Topaz	28.12 ±3.60abcd	36.50 ±10.51ab	2.45 ±0.25a	12.13 ±0.84a	5.74 ±0.34abc	0.63 ±0.22ab
Roter Boskoop	22.68 ±7.60bcd	43.99 ±2.81a	1.97 ±0.07a	10.06 ±1.03a	5.42 ±1.19abcd	1.64 ±1.27ab
Braeburn	15.51 ±2.57bcd	23.79 ±2.58ab	2.15 ±0.25a	11.82 ±1.75a	6.53 ±0.87ab	1.37 ±0.78ab
Cox Orange	16.48 ±2.83bcd	36.45 ±7.83ab	2.18 ±0.28a	10.66 ±4.01a	2.42 ±0.66d	0.76 ±0.23ab
Elstar	34.42 ±16.10ab	29.71 ±2.13ab	2.44 ±1.56a	8.30 ±2.57a	3.90 ±2.21abcd	0.63 ±0.26ab
Steir. Maschanzker	28.57 ±6.65abcd	29.26 ±14.54ab	2.66 ±0.21a	12.94 ±1.85a	3.28 ±0.37bcd	0.72 ±0.07ab
Gravensteiner	18.91 ±1.65bcd	30.12 ±6.28ab	2.93 ±0.76a	13.97 ±5.84a	4.10 ±0.89abcd	2.11 ±0.75a
Ilzer Rosenapfel	32.55 ±1.69abc	42.18 ±17.74a	1.62 ±0.25a	12.81 ±1.11a	2.48 ±0.56cd	1.11 ±0.57ab
Goldparmäne	44.07 ±9.41a	27.31 ±4.79ab	1.59 ±0.20a	11.00 ±1.21a	3.55 ±1.34abcd	0.42 ±0.04b
Roter Berlepsch	11.34 ±2.69d	14.74 ±6.60b	1.75 ±0.20a	7.55 ±1.61a	4.66 ±0.96abcd	0.38 ±0.09b
Winterbanane	28.03 ±9.40abcd	36.17 ±3.98ab	2.44 ±0.10a	11.36 ±2.56a	4.37 ±0.43abcd	1.46 ±0.42ab
Rubinette	17.97 ±2.41bcd	15.96 ±1.16b	1.98 ±0.13a	11.29 ±0.99a	6.82 ±0.71a	0.44 ±0.05b
Kronprinz Rudolf	13.07 ±1.68cd	19.80 ±7.64ab	1.76 ±0.25a	13.55 ±1.73a	6.10 ±2.42ab	0.36 ±0.07b

B

Cultivar	Untreated Juice	Treated Juice	Pomace
Topaz	28.12 ±3.60a	36.50 ±10.51a	2.45 ±0.25b
Roter Boskoop	22.68 ±7.60b	43.99 ±2.81a	1.97 ±0.07c
Braeburn	15.51 ±2.57b	23.79 ±2.58a	2.15 ±0.25c
Cox Orange	16.48 ±2.83b	36.45 ±7.83a	2.18 ±0.28c
Elstar	34.42 ±16.10a	29.71 ±2.13a	2.44 ±1.56b
Steir. Maschanzker	28.57 ±6.65a	29.26 ±14.54a	2.66 ±0.21b
Gravensteiner	18.91 ±1.65b	30.12 ±6.28a	2.93 ±0.76c
Ilzer Rosenapfel	32.55 ±1.69a	42.18 ±17.74a	1.62 ±0.25b
Goldparmäne	44.07 ±9.41a	27.31 ±4.79b	1.59 ±0.20c
Roter Berlepsch	11.34 ±2.69ab	14.74 ±6.60a	1.75 ±0.20b
Winterbanane	28.03 ±9.40a	36.17 ±3.98a	2.44 ±0.10b
Rubinette	17.97 ±2.41a	15.96 ±1.16a	1.98 ±0.13b
Kronprinz Rudolf	13.07 ±1.68ab	19.80 ±7.64a	1.76 ±0.25b

C

Cultivar	Peel	Flesh	Seeds
Topaz	12.13 ±0.84a	5.74 ±0.34b	0.63 ±0.22c
Roter Boskoop	10.06 ±1.03a	5.42 ±1.19b	1.64 ±1.27c
Braeburn	11.82 ±1.75a	6.53 ±0.87b	1.37 ±0.78c
Cox Orange	10.66 ±4.01a	2.42 ±0.66b	0.76 ±0.23b
Elstar	8.30 ±2.57a	3.90 ±2.21ab	0.63 ±0.26b
Steir. Maschanzker	12.94 ±1.85a	3.28 ±0.37b	0.72 ±0.07b
Gravensteiner	13.97 ±5.84a	4.10 ±0.89b	2.11 ±0.75b
Ilzer Rosenapfel	12.81 ±1.11a	2.48 ±0.56b	1.11 ±0.57b
Goldparmäne	11.00 ±1.21a	3.55 ±1.34b	0.42 ±0.04c
Roter Berlepsch	7.55 ±1.61a	4.66 ±0.96b	0.38 ±0.09c
Winterbanane	11.36 ±2.56a	4.37 ±0.43b	1.46 ±0.42b
Rubinette	11.29 ±0.99a	6.82 ±0.71b	0.44 ±0.05c
Kronprinz Rudolf	13.55 ±1.73a	6.10 ±2.42b	0.36 ±0.07c

Supplementary Table S6. Vitamin C and dehydroascorbic acid content for the processed apple juice. The HILIC-MS/MS measurements were performed in triplicates. Results are shown as mg of ascorbic acid or mg of dehydroascorbic acid per L of juice. <LOQ: below limit of quantification.

**Cultivar**	**Vitamin C (mg/L)**	**dehydroascorbic acid (mg/L)**
Topaz	<LOQ	66.3 ±2.4
Roter Boskoop	<LOQ	41.8 ±4.9
Braeburn	<LOQ	62.7 ±4.0
Cox Orange	<LOQ	28.6 ±2.1
Elstar	<LOQ	30.9 ±4.1
Steir. Maschanzker	<LOQ	44.5 ±2.0
Gravensteiner	11.3 ±0.7	<LOQ
Ilzer Rosenapfel	<LOQ	78.6 ±6.2
Goldparmäne	<LOQ	70.9 ±4.4
Roter Berlepsch	<LOQ	125.6 ±0.2
Winterbanane	<LOQ	76.5 ±5.0
Rubinette	211.5 ±5.7	110.2 ±4.9
Kronprinz Rudolf	<LOQ	35.1 ±3.5

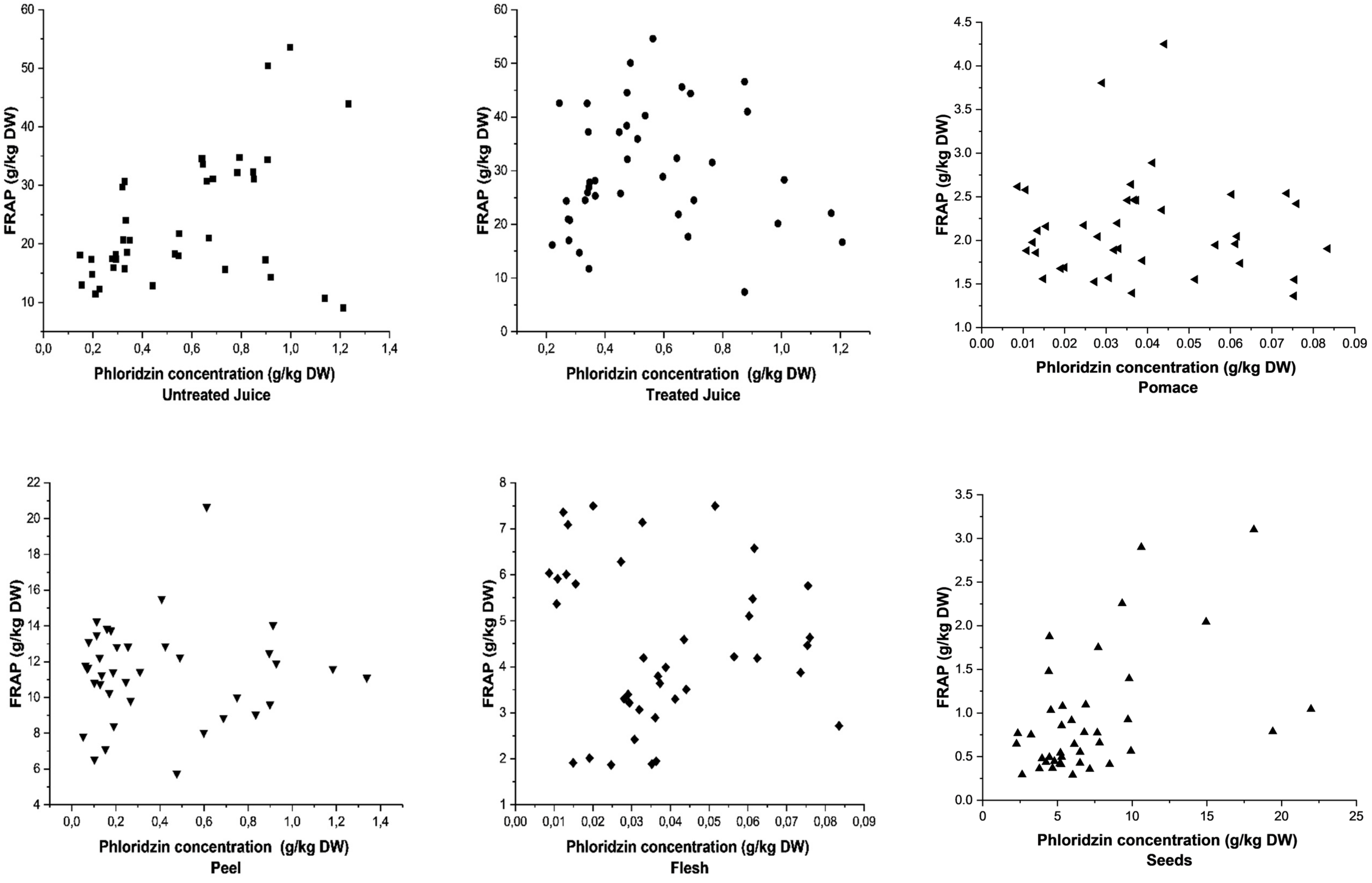

Supplementary Figure S1. Juxtaposition of antioxidant activity (FRAP) and the concentration of phloridzin in all processed juice and solid apple parts sample.

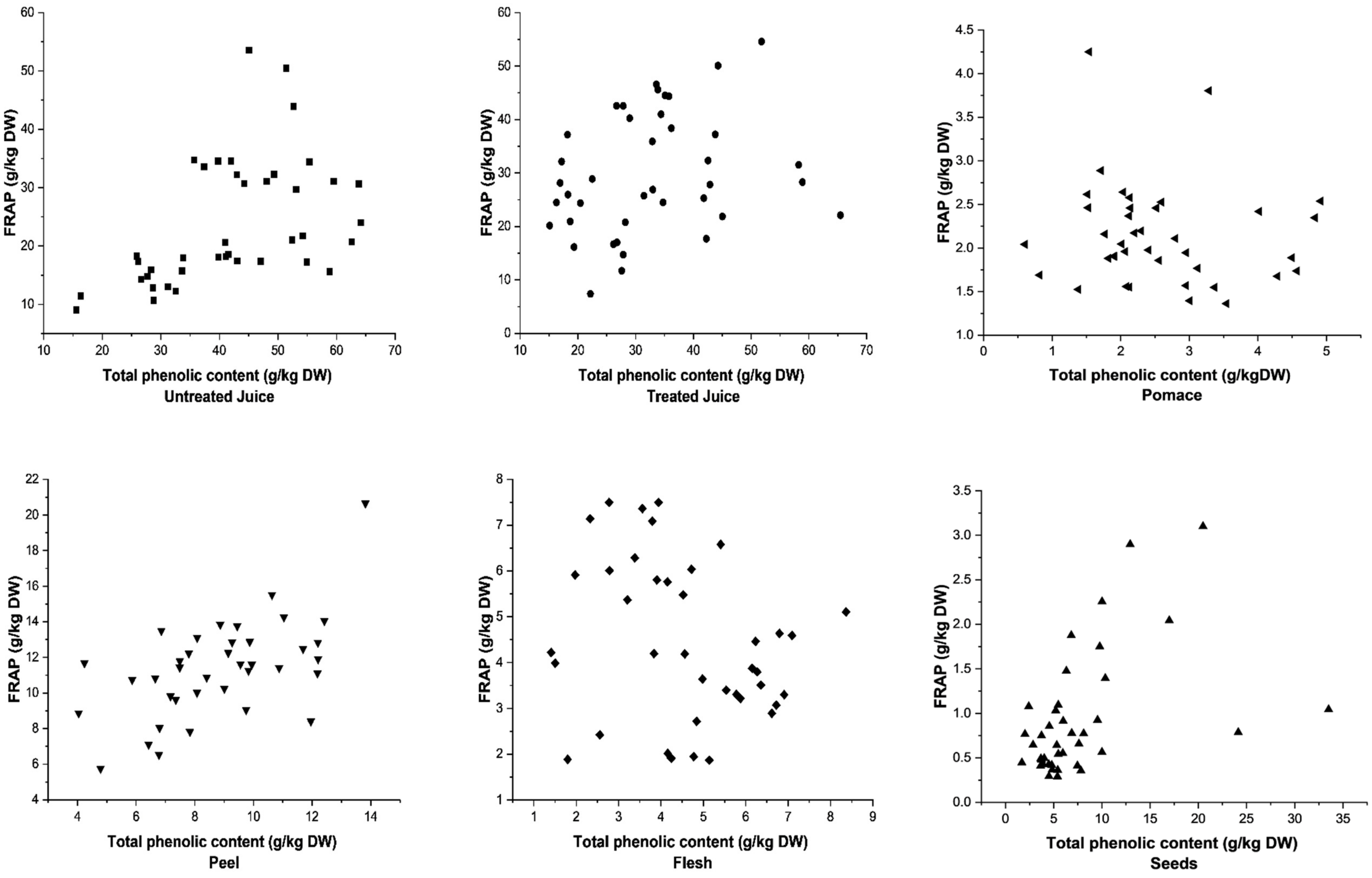

Supplementary Figure S2. Juxtaposition of antioxidant activity (FRAP) and the total phenolic content in all processed juice and solid apple parts sample.

References

1. Fischer, M. *Farbatlas Obstsorten*; Verlag Eugen Ulmer: Stuttgart, Germany, 2003.
2. Ilzer Rose Apple. Available online: https://www.pur-apfel.at/sorten/ilzer-rosenapfel (accessed on 12 September 2024).
3. Hartmann, W. *Farbatlas Alte Obstsorten*; Verlag Eugen Ulmer: Stuttgart, Germany, 2000.
4. Styrian Maschanzker. Available online: https://austria-forum.org/af/Heimatlexikon/Steirischer_Maschanzker (accessed on 12 September 2024).
